# Case report: bilateral Mooren ulcer in association with hepatitis C

**DOI:** 10.1186/s12886-017-0633-x

**Published:** 2017-12-06

**Authors:** Vesa Aaltonen, Mari Alavesa, Laura Pirilä, Eija Vesti, Mohammad Al-Juhaish

**Affiliations:** 1 Department of Ophthalmology, Turku University Hospital, PO Box 52, FIN-20521 Turku, Finland; 2 Department of Ophthalmology, University of Turku, FIN-20014 Turku, Finland; 3 Department of Rheumatology, Division of Medicine, Turku University Hospital, Box 52, FIN-20521 Turku, PO Finland; 4 Department of Internal Medicine, University of Turku, FIN-20014, Turku, Finland

**Keywords:** Mooren ulcer, Keratitis, Hepatitis C, Immunomodulation, Immunosuppression

## Abstract

**Background:**

Mooren ulcer has been considered as an idiopathic autoimmune keratitis. However, it has been in some cases suggested to be associated with hepatitis C, although the evidence is very vague.

**Case presentation:**

We present a case of a man who was diagnosed with a primary Mooren ulcer in his right eye. The eye became blind despite of intensive treatment with local medications and extensive surgical procedures. After 10 years, the patient was diagnosed with the same disease, now in his left, previously healthy eye. There was no history that would suggest a secondary Mooren ulcer, but a chronic hepatitis C infection was detected. Treatment was targeted against hepatitis C (ribavirin and interferon) in addition to immunosuppressive medical and surgical treatment which resulted in a full and more than 6 years lasting remission of the disease.

**Conclusions:**

Whether the immunomodulatory and immunosuppressive medication against hepatitis C was the key reason for the good results in the treatment of the second eye, remains elusive. The causality of hepatitis C with respect to the pathogenesis of Mooren ulcer on this patient remains open, but should be considered as one of the possible etiological factors of the disease.

## Background

Mooren ulcer is a rare, unilateral or bilateral, painful and chronic corneal ulcer. It begins with an inflammation of conjunctiva and episclera and a peripheral corneal ulcer near the limbus. The ulcer then progresses circumferentially and centrally and may destroy the corneal stroma leaving behind only a thin fibrovascular membrane and an intact Descemet’s membrane. As a devastating complication, Mooren ulcer frequently results in a perforation of the eye [1]. The etiological factors leading to Mooren ulcer are not understood. Corneal surgery, trauma, infection, and also parasitic infections and hepatitis C has been suggested in the literature to take part in the cascade leading to Mooren ulcer [1–8]. However, there is clear evidence that suggests that Mooren ulcer is an autoimmune disorder, directed against the cornea [1]. In general, the medical treatment of Mooren ulcer typically includes first topical and systemic immunosuppression. Additional surgical approaches often include a conjunctival excision, but different types of keratoplasty and keratotomy have also been used, together with different types of patches.

## Case presentation

**Fig. 1:**
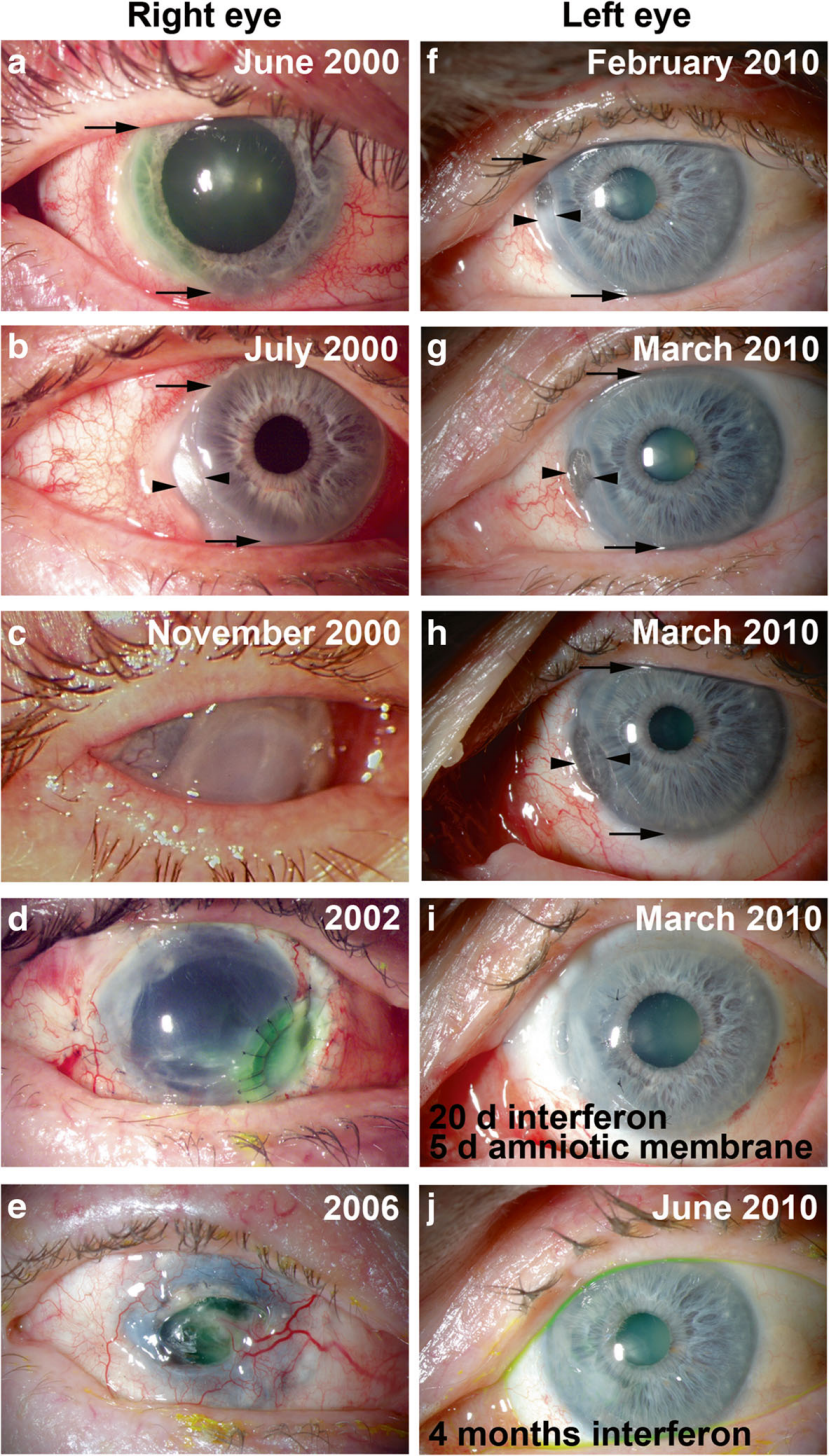
Clinical photographs of the (**a**-**e**) right and (**f**-**j**) left eye of the same patient showing developing Mooren ulcer (**a**-**b** and **f**-**h**) and the end results after different treatment strategies for right (**c**-**e**) and left (**i**-**j**) eyes. Arrows point to the peripheral ulcer ends and arrowheads point to deeper ulcers threatening to perforate the cornea

The patient is a 72 years old male with a previous medical history showing dermatitis herpetiformis which, with gluten-free diet remained asymptomatic. He had been diagnosed with a Mooren ulcer in his right eye in 2000 (Fig. 1a) with devastating end results. At that time, an internist was consulted but no etiological explanations were found. Initial medical interventions for the right eye included p.o. and topical cyclosporine, p.o. prednisolone, chloramphenicol-hydrocortisone caproate drops and aprotinin drops*.* Since the ulcer continued to progress (Fig. 1b), a tarsorrhaphy and subsequently a conjunctival flap were performed to cover the ulcer. As these failed (Fig. 1c), a corneoscleral transplantation combined with cataract extraction was performed 6 months after the first symptoms. A fistula developed postoperatively, which was operated a total of 29 times including re-suturations and different types of patches (tissue glue, amniotic membrane and scleral transplants) during early 2002. Later in 2002, a sterile ulcer and a spontaneous perforation developed and a lamellar corneal transplantation was performed without complications (Fig. 1d). However, in 2004, the patient’s right eye spontaneously perforated which was treated with re-suturation and amniotic membrane transplantation. The end-stage was phthisis and visual acuity (VA) of only faint sense of light in March 2006 (Fig. 1e).

The patient arrived to the hospital again in January 2010 reporting redness and pain in his previously healthy left eye. He had VA 0.7 and biomicroscopy showed an oval ulcer with sharp borders in the nasal cornea near limbus, with slight local chemosis in the conjunctiva of the left eye. Bacterial cultures were negative, and there was no preceding trauma. Despite of the patient’s medical history, a marginal ulcer was diagnosed. Dexamethasone-chloramphenicol (D-C) drops and Chloramphenicol ointment were prescribed. Plasma aspartate aminotransferase and alanine aminotransferase were slightly elevated. After 3 weeks from the onset of the symptoms, the ulcer had progressed circumferentially and centrally as a crescent shaped ulcer with steep central borders. In the center of the ulcer there was another deeper ulcer with steep margins and an epithelial defect. The stroma had melted to the level of Descemet membrane and limbus. There was conjunctival inflammation, but the sclera was intact. A senior ophthalmologist reached the diagnosis of Mooren ulcer (Fig. 1f). The diagnosis of Mooren ulcer was established by elimination of other etiologies for peripheral ulcerative keratitis (PUK). There was no evidence of microbial etiology, trauma, or exposure keratopathy and no findings suggesting rosacea (blepharitis or rash). Terrien marginal degeneration was excluded since the location of that degeneration is in superior cornea, ulcus does not exist, and it does not progress centrally. Also, in Fuchs superficial marginal keratitis, the epithelium is not affected. Pellucid marginal degeneration is located in inferior cornea and, like in Senile furrow degeneration, there is no inflammation. Rheumatoid arthritis may cause similar findings as in the presented patient, but sclera was not involved, and there was no evidence of this condition in clinical evaluation. In addition, other PUK’s typically spare the limbus, which is involved in Mooren ulcer as was in the presented case also. Other systemic etiologies were ruled out by clinical evaluation by an internist and negative laboratory results. Systemic conditions associated with PUK, include infections (tuberculosis, syphilis, gonorrhea, borreliosis, bacillary dysentery, herpes zoster, AIDS, and hepatitis C), rheumatoid arthritis, systemic lupus erythematosus, Wegener granulomatosis, polyarteritis nodosa, relapsing polychondritis, scleroderma, progressive systemic sclerosis, Sjögren syndrome, Beçhet’s disease, sarcoidosis, α1-antitrypsin deficiency, malignancy and inflammatory bowel disease [9, 10].

The treatment continued with D-C drops and artificial tears. Unfortunately the patient did not come to the controls. After 5 weeks from the onset the patient eventually showed up. There was a large curved vertical ulcer in the nasal cornea which included a deep 2.5 × 1.7 mm ulcer extending to the Descemet membrane (Fig. 1g).

A conjunctival resection was performed during the same day. Further therapy included a therapeutical contact lens, 0.05% cyclosporine drops q6h., D-C drops q6h., ofloxacin drops q6h., methotrexate 10 mg and folic acid 5 mg weekly, and i.v. prednisolone 500 mg for three days, and subsequently p.o. prednisolone 60 mg daily. As a result of cortisone treatment the patient developed hyperglycemia which was treated with s.c. Detemir and Aspart Insulins. At this point, further therapy with systemic adalimumab or infliximab was considered.

As described above, the autoimmune diseases were excluded by clinical examination and serological tests. However, the patient’s current laboratory results showed an active hepatitis C infection (quantitative RNA 1.25 × 10^6^ IU/ml, genotype 1b). The source of the infection was considered to be a blood transfusion during a surgery in 1977. The possible association of hepatitis C with Mooren ulcer was ignored in 2000 when the patient’s right eye was diagnosed and treated. Thus, the patient was not tested for hepatitis C at that time.

As the hepatitis C was confirmed, a gastroenterologist was consulted. He did not suggest treatment of hepatitis C because of high age of the patient and lack of typical hepatitis symptoms. However, since Hepatitis C has been considered as an etiological factor in Mooren ulcer, we decided to discontinue methotrexate which the patient had had only one weekly dose, and asked gastroenterologist to start the treatment for hepatitis C. This took place 6.5 weeks after onset of the symptoms in the left eye. The treatment included peginterferon alfa-2b (PegIntron) 100 μg weekly and ribavirin (Copegus) 1000 mg derived into two doses (400 mg + 600 mg) daily, which is a normal regime used for Hepatitis C treatment. Therapeutic contact lens, cyclosporine drops, D-C drops and ofloxacine drops were continued and oral prednisolone was reduced 5 mg every 4 days. In the control after 12 days from the first dose of interferon the nasal ulcer was deeper (Fig. 1h). During the same day, an amniotic membrane was sutured to cover the ulcer, and the oral prednisolon was raised to 60 mg and later tapered more slowly. As a side effect of the treatments, the patient developed neutropenia which was treated with pegfilgrastim (Neulasta). The patient did not respond fully to the combination treatment with interferon and ribavirin since after 24 weeks of treatment the hepatitis C quantitative RNA result was 0.36 × 10^6^ IU/ml (in the beginning of treatment was 1.25 × 10^6^ IU/ml). However, the above mentioned procedures rapidly and permanently stabilized the ulcer (Fig. 1i and j) and the systemic and topical drugs could be terminated. After 6 years of follow up, the eye is still healthy.

## Discussion and conclusions

In conclusion, the only major difference in the initial treatment strategies between the patient’s right and left eye was interferon combined with ribavirin. The treatment of the right eye was unsuccessful despite of exhaustive efforts. In contrast, the left eye rapidly and permanently recovered with the treatment. The hepatitis C persisted, albeit with reduced viral count. Previously, Wilson et al. described two patients with Mooren type ulceration in association of chronic hepatitis C infection. In both patients, the corneal disease improved when interferon alpha-2b treatment for chronic active hepatitis. In one of these patients, the corneal disease worsened when the treatment was discontinued, and improved again when the treatment continued again [3]. Furthermore, Moazami et al. described a patient with Mooren ulcer and hepatitis C and showed an improvement of the ulcer which correlated with normalization of the patient’s liver function test [4]. Interestingly, Erdem et al. reported two Mooren ulcer patients who were treated with topical Interferon alpha-2a, and the lesions resolved within 10 days without recurrence. It was not reported whether the patients had Hepatitis C or not [11]. This result may suggest that it is possible that the positive improvement may be due to direct immunomodulation by interferon. However, this does not exclude the possibility of association of Hepatitis C with Mooren ulcer, or improvement of the lesion with the Hepatitis C treatment. Thus, the causality of hepatitis C with respect to the pathogenesis of Mooren ulcer in our patient remains open but possible. Since autoimmune mechanisms may be involved in the pathogenesis of Mooren ulcer [6, 7], the successful treatment results were possibly directly due to immunomodulatory properties of interferon combined with immunosuppression with cortisone and cyclosporine.

## Abbreviations

D-C: Dexamethasone-chloramphenicol
RNA: Ribonucleic acid
VA: Visual acuity
